# Mental Health in Cystic Fibrosis in the Modulator Era: Epidemiology, Prognostic Significance, and Therapeutic Implications

**DOI:** 10.3390/jcm15103953

**Published:** 2026-05-20

**Authors:** Maryam M. Almulhem, Rayan A. Siraj

**Affiliations:** Department of Respiratory Therapy, College of Applied Medical Sciences, King Faisal University, Al-Ahsa 31982, Saudi Arabia; rsiraj@kfu.edu.sa

**Keywords:** cystic fibrosis, mental health, depression, anxiety, CFTR modulators, quality of life, treatment adherence

## Abstract

Individuals with cystic fibrosis (CF) face significant treatment burdens, and as life expectancy has increased, there is growing emphasis on their psychosocial well-being. Prevalence data indicate that approximately one-quarter to one-third of individuals with CF and their caregivers experience clinically significant anxiety or depression. Specifically, pooled global estimates report an anxiety prevalence of 24.9% (95% CI: 20.8–28.9%) and depression prevalence of 13–33% in adults with CF, with caregivers experiencing even higher rates (anxiety: 35–38%; depression: 20–35%). Depression is independently associated with a nearly twofold increase in mortality risk and substantially higher healthcare costs, underscoring its prognostic significance. These mental health comorbidities are consistently associated with reduced treatment adherence, diminished quality of life, increased healthcare utilisation, and decreased survival. Accordingly, psychological well-being has emerged as a key patient outcome that directly shapes engagement with care and the effectiveness of long-term CF management. International CF guidelines now recommend routine mental health screening within multidisciplinary care frameworks. Evidence-based interventions include cognitive–behavioural therapy (CBT), which is endorsed as a primary treatment, although access remains limited, and stepped-care pharmacotherapy, primarily selective serotonin reuptake inhibitors (SSRIs), for moderate to severe symptoms. Telemedicine and other digital health approaches have expanded access to psychological support, with remote CBT and online programmes demonstrating feasibility and symptom improvement during the COVID-19 pandemic and beyond. The advent of CFTR modulator therapies has significantly altered clinical outcomes, enabling many patients to achieve improved lung function and daily functioning. Nevertheless, mental health challenges persist, as individuals navigate new identity shifts and anxieties despite enhanced physical health. The implementation of mental healthcare remains inconsistent; while screening rates have increased, timely follow-up and integrated psychosocial support are frequently insufficient across care centres. This narrative review highlights the ongoing need to integrate mental health management into CF care to optimise adherence, patient outcomes, and long-term survival in the current therapeutic landscape.

## 1. Introduction

Cystic fibrosis (CF) is a chronic, autosomal recessive genetic disorder caused by mutations in the cystic fibrosis transmembrane conductance regulator (CFTR) gene [1,2]. These mutations lead to dysfunctional or absent ion transport, resulting in thick, dehydrated secretions that obstruct ducts and airways, creating a milieu for chronic inflammation and progressive organ damage [3].

Although CF is primarily recognised as a pulmonary disease, its consequences extend far beyond the respiratory system. Individuals with CF endure a substantial daily treatment burden and face ongoing medical, nutritional, and social challenges, often from early childhood into adulthood [4,5]. As life expectancy has improved, there is increasing recognition of the psychosocial consequences of living with CF, particularly the high prevalence of anxiety and depression. These mental health comorbidities can negatively affect treatment adherence, disease outcomes, overall quality of life, and even mortality, highlighting the need for integrated, multidisciplinary care [6]. Importantly, much of the existing evidence describing these associations was generated prior to the widespread availability of CFTR modulator therapies, and therefore requires reinterpretation within the current therapeutic context.

These psychosocial challenges, spanning anxiety related to disease progression and invasive procedures, depressive symptoms driven by treatment burden and social disruption, caregiver distress, and difficulties navigating developmental transitions, are not incidental to disease management but directly shape it. They are associated with reduced adherence to complex daily regimens, increased healthcare utilisation, accelerated pulmonary decline, and increased mortality risk [6,7], as detailed in the sections that follow.

Importantly, mental health outcomes in the CF are increasingly recognised as central patient outcomes that shape engagement with care, treatment adherence, and the effectiveness of long-term disease management [8].

The therapeutic landscape of cystic fibrosis has changed substantially with the introduction of highly effective CFTR modulator therapies. Treatments such as elexacaftor–tezacaftor–ivacaftor (ETI) have improved lung function, reduced pulmonary exacerbations, and extended survival for many individuals with CF [9,10]. As a result, CF is increasingly understood as a chronic condition with evolving long-term psychosocial implications. In this changing therapeutic context, understanding mental health outcomes in the modulator era has become increasingly important, as improvements in physical health do not necessarily eliminate psychological vulnerability [8,11]. While recent studies have begun to explore mental health outcomes following the introduction of CFTR modulators, existing literature often focuses on specific clinical or psychosocial domains in isolation. While recent studies have begun to explore mental health outcomes following the introduction of CFTR modulators, existing literature often focuses on specific clinical or psychosocial domains in isolation. Moreover, much of the current evidence base remains rooted in pre-modulator populations, necessitating careful reinterpretation in light of evolving clinical realities. Unlike prior reviews, this manuscript provides an integrated synthesis across epidemiology, clinical correlates, screening strategies, therapeutic interventions, special populations, and implementation challenges, while explicitly reinterpreting existing evidence within the context of highly effective CFTR modulator therapy. Accordingly, this narrative review explores the prevalence and impact of anxiety and depression in cystic fibrosis, their clinical correlates, screening and treatment approaches, challenges in implementation, and emerging strategies for integrating mental health into routine CF care within the context of the modulator era.

## 2. Epidemiology, Prevalence, and Incidence of Depression and Anxiety in Cystic Fibrosis

Depression and anxiety are clinically defined psychological conditions that are increasingly recognised as prevalent comorbidities in CF. Depression is commonly conceptualised as a mood disorder characterised by persistent low mood and/or loss of interest or pleasure, accompanied by cognitive and behavioural changes that impair social or occupational functioning [12]. Anxiety is typically defined as a state of excessive apprehension, fear, or uncertainty that disrupts normal psychological and physiological functioning [13]. In individuals with CF, anxiety may present in both generalised and context-specific forms. Procedural anxiety, related to medical or surgical procedures, may be under-recognised despite repeated exposure to healthcare interventions. Importantly, the majority of epidemiological data describing depression and anxiety in cystic fibrosis has been generated in pre-modulator populations and should therefore be interpreted within this temporal context.

Within this conceptual framework, epidemiological studies, largely conducted in the pre-modulator era, consistently demonstrate elevated prevalence of both depression and anxiety among people with CF and their caregivers. Psychological distress affects a substantial proportion of patients and families across age groups and care contexts [6,7]. Prevalence estimates across CF populations are summarised in Table 1. Although these ranges reflect variability in study design and measurement, they collectively indicate that psychological distress is typical rather than exceptional in CF.

Extensive pooled analyses and multinational screening initiatives—primarily reflecting pre-modulator populations—confirm these findings, suggesting that approximately one-quarter to one-third of individuals affected directly or indirectly by CF experience clinically relevant symptoms of anxiety or depression at any given time [7].

Findings from international screening programmes have further shown that rates of anxiety and depression among people with CF and their caregivers are markedly higher than those observed in community reference samples [6]. Anxiety and depression were frequently comorbid across both patient and caregiver groups, and family-based analyses demonstrated clustering of psychological symptoms within households, with adolescents substantially more likely to report elevated symptoms when a parent caregiver also screened positive.

Global prevalence has also been examined using meta-analytic approaches that permit subgroup exploration. In a systematic review and meta-analysis including 26 studies and 9766 individuals with CF, the pooled global prevalence of anxiety, after correction for publication bias, was estimated at 24.9% (95% CI: 20.8–28.9). Subgroup analyses suggested modest regional variation, with anxiety prevalence lowest in North America (23.6%) and highest in Europe (26.8%) [7]. In contrast, depressive symptoms demonstrated an inverse pattern, with the highest pooled prevalence reported in North America (18.7%) and lower estimates observed in European cohorts (13.3%). Notably, confidence intervals were wide and between-study heterogeneity remained substantial, indicating that apparent regional differences should be interpreted cautiously [7]. Importantly, these pooled estimates are derived from studies conducted prior to widespread CFTR modulator use and may not fully capture emerging patterns in modulator-treated populations.

In contrast to the expanding prevalence literature, incidence of anxiety and depression remains poorly characterised [6,7]. Cross-sectional screening studies dominate the epidemiological evidence base, and population-based incidence estimates for anxiety and depression in CF are largely unavailable [7]. Longitudinal data capable of distinguishing transient psychological distress from persistent or recurrent disorders across the CF lifespan remain limited, constraining the understanding of symptom trajectories over time [15]. This limitation is particularly relevant in the modulator era, where changes in disease trajectory may alter the onset, persistence, and recurrence of psychological symptoms.

Age-related patterns further illustrate the complexity of CF mental health epidemiology. Synthesised evidence suggests that depressive symptoms are generally less prevalent in adolescents than in adults, whereas anxiety symptoms are already prominent during adolescence and remain at comparable levels into adulthood [16]. Evidence regarding younger children with CF is notably limited. Paediatric-focused reviews examining anxiety in children aged 6–12 years and their parents suggest that anxiety may be the most prevalent mental health concern in this age group [16]; however, the absence of studies focusing exclusively on this developmental stage and the use of heterogeneous assessment approaches limit the precision of prevalence estimates and understanding of early trajectories. Whether these developmental patterns persist or shift in the context of improved clinical stability associated with CFTR modulator therapy remains unclear.

Finally, cross-cultural variation in reported prevalence has been observed across multinational studies and global meta-analyses [17]. However, differences between countries and regions frequently parallel variation in screening instruments, cut-off thresholds, and assessment practices. The lack of formal evaluation of measurement equivalence across cultures and developmental stages complicates the interpretation of cross-national prevalence estimates and limits confidence in direct comparisons.

Beyond cross-cultural variation, several broader methodological constraints limit the interpretation of prevalence estimates across the CF literature. Most prevalence data derive from specialist CF centre cohorts [7]. As a result, they may not fully represent patients receiving care in community settings or those with more limited healthcare access. This introduces a potential source of selection bias that has received relatively limited formal attention [17]. In addition, substantial heterogeneity arises from variation in assessment tools, diagnostic thresholds, and study designs across populations and settings. Future research should therefore prioritise the adoption of standardised, cross-culturally validated measurement approaches within CF registries and multinational research programmes [18]. Harmonisation of assessment tools and reporting thresholds across studies would improve the comparability of prevalence estimates and strengthen the evidence base for targeted psychosocial interventions. These methodological limitations may further constrain the interpretation of prevalence estimates in the modulator era, where evolving clinical characteristics introduce additional sources of heterogeneity.

### Mental Health in the Modulator Era

The introduction of highly effective CFTR modulator therapy, particularly ETI, has raised important questions regarding how the epidemiology of depression and anxiety in cystic fibrosis may evolve. Emerging evidence from the modulator era presents a heterogeneous and at times conflicting picture [19]. Registry-based analyses suggest that the overall prevalence of depression and anxiety following ETI initiation has remained broadly consistent with trends observed prior to modulator availability, indicating no substantial population-level shift in prevalence to date [19]. However, individual-level responses appear more variable. Several cohort studies report improvements in depressive and anxiety symptoms for many individuals after ETI initiation, while a smaller subgroup experiences new-onset or worsening psychological symptoms despite physical gains [20,21]. Factors such as pre-existing psychiatric diagnoses and psychosocial vulnerability may influence these trajectories [11,19]. Collectively, these findings suggest that the mental health landscape in CF during the modulator era is evolving rather than uniformly improving, and the current prevalence estimates likely reflect a transitional period. However, existing evidence remains constrained by short follow-up durations and considerable heterogeneity in study design, precluding definitive conclusions regarding long-term mental health trajectories in ETI-treated populations and underscoring the need for continued epidemiological surveillance [15].

## 3. Clinical Correlates and Health Outcomes Associated with Depression and Anxiety in Cystic Fibrosis

### 3.1. Associations with Pulmonary Function and Disease Severity

Cross-sectional studies consistently demonstrate inverse associations between depressive symptoms and lung function in adolescents and adults with CF [22]. Across cohorts, higher levels of depression are associated with lower FEV_1_, whereas anxiety appears more closely related to respiratory symptom severity rather than objective pulmonary measures [23]. Together, these patterns suggest that depression, more than anxiety, is consistently linked to impaired pulmonary function in CF.

More informative evidence is provided by prospective analyses examining changes in lung function over time. A longitudinal study conducted within the framework of the international TIDES program followed 473 adolescents and adults with CF in Germany over two years [23]. Baseline depressive symptoms, assessed using the Hospital Anxiety and Depression Scale, significantly interacted with baseline lung function to predict subsequent change in FEV_1_. Notably, individuals with clinically relevant depressive symptoms and relatively preserved lung function at baseline experienced the most significant decline in FEV_1_ during follow-up. In contrast, patients without depressive symptoms and poorer baseline lung function demonstrated relative stability or modest improvement.

This longitudinal pattern challenges the notion that depression merely reflects advanced disease severity. While worsening lung function and symptom burden may contribute to psychological distress, these findings indicate that depressive symptoms can precede and predict subsequent pulmonary decline, particularly in earlier stages of disease. Such observations support the possibility of a bidirectional or reciprocal relationship between mental health and pulmonary outcomes in CF, although causal mechanisms cannot be definitively established [22,23]. However, whether this bidirectional relationship persists with similar magnitude in the context of improved clinical stability associated with CFTR modulator therapy remains uncertain.

Despite these advances, important limitations remain. Most studies rely on symptom screening instruments rather than diagnostic assessment, and adjustment for key clinical confounders varies across analyses [22,23]. In addition, follow-up periods remain short relative to the lifelong course of CF, limiting the understanding of long-term pulmonary trajectories [23]. These limitations are particularly relevant in the modulator era, where altered disease trajectories may modify the relationship between psychological factors and pulmonary outcomes.

It is important to contextualise these pre-modulator findings within the current therapeutic landscape. The TIDES study and the cross-sectional studies cited above were conducted largely before or during the early availability of elexacaftor/tezacaftor/ivacaftor (ETI), in populations carrying substantially greater disease burden than many patients experience today. Since ETI approval, marked improvements in lung function, nutritional status, and daily functioning have been reported, and several studies document concurrent reductions in depression and anxiety symptoms following treatment initiation [11,20]. However, the mental health response to ETI is heterogeneous. While many patients improve to normative psychological levels within 12–18 months, a subgroup develops new-onset or persistent symptoms despite physical gains, potentially reflecting identity disruption, grief over illness-related roles, or anxiety about long-term treatment durability [8,15]. These findings collectively suggest that improvements in physical health do not uniformly translate into improvements in psychological well-being. Whether the bidirectional relationship between depression and pulmonary decline documented in the pre-modulator era operates with the same magnitude and directionality in ETI-treated populations, where lung function trajectories are fundamentally altered, remains an important and unanswered question that future longitudinal research must address [8,15].

### 3.2. Health Related Quality of Life

Health-related quality of life (HRQoL) is a central outcome in CF, reflecting the cumulative effects of physical disease burden, treatment demands, and psychosocial functioning [22,24]. Although lung function remains a key indicator of disease severity, its association with HRQoL is often modest [22]. This has prompted increased attention to psychological factors—particularly anxiety and depression—as determinants of patient-reported well-being [24].

Evidence from adult CF cohorts consistently demonstrates that depressive symptoms are strongly associated with impaired HRQoL, independent of pulmonary disease severity. In a well-characterised adult sample of 76 patients with CF, approximately 30% screened positive for clinically relevant depressive symptoms. While depressive symptoms were modestly correlated with lung function, their association with HRQoL persisted after stratification by lung function [22]. Patients with depressive symptoms reported significantly poorer HRQoL across the physical, emotional, and social domains regardless of whether lung function was preserved or impaired.

Similar findings have been reported in other adult CF cohorts. In a clinic-based study of 121 adults, 33% screened positive for anxiety symptoms and 17% for depressive symptoms. Both anxiety and depression were associated with impairment across multiple CF-specific HRQoL domains including physical functioning, emotional functioning, social functioning, chest symptoms, and treatment-related concerns [25]. Depressive symptoms showed stronger associations with lower lung function and overall HRQoL, whereas anxiety was more closely linked to subjective respiratory symptom burden and interpersonal functioning [25]. These patterns suggest overlapping yet distinct pathways through which anxiety and depression influence HRQoL in CF.

Additional insight is provided by studies examining coping styles. In a study of 30 adult CF patients, 40% met criteria indicating risk for anxiety and/or depression [26]. Individuals in the anxiety-risk group reported lower HRQoL scores in vitality, emotional functioning, and role limitations, while those with depressive symptoms demonstrated poorer emotional functioning and greater role limitations. Anxiety and depression scores were identified as significant predictors of emotional HRQoL, independent of other assessed variables. Maladaptive coping strategies, particularly avoidance, were more common among individuals at risk, whereas acceptance was the most frequently reported coping strategy overall [26].

Interpretation of these findings requires caution. HRQoL instruments include emotional and social domains that overlap conceptually with measures of anxiety and depression, raising the possibility of shared variance [26]. In addition, most studies employ cross-sectional designs, limiting insight into the temporal relationship between psychological distress, coping strategies, and HRQoL trajectories [22,25].

Following ETI initiation, improvements in physical and respiratory HRQoL domains have been widely reported [27,28]. However, emotional and social HRQoL outcomes appear more variable. A prospective longitudinal study evaluating ETI effects at 1, 3, and 6 months found significant improvements in CFQ-R respiratory and physical domains, yet emotional functioning did not improve consistently across all timepoints, and a subgroup continued to report persistent depressive or anxiety symptoms [27]. In addition, a mixed-methods study reported that while CFQ-R physical and respiratory scores were relatively high in ETI-treated adults, emotional and treatment-related domains remained lower, suggesting that psychological burden may persist despite improvements in physical health [28]. These findings underscore the importance of continued psychological monitoring in the modulator era, as gains in physical health do not uniformly translate into improvements in emotional well-being.

### 3.3. Impact of Depression on Treatment Adherence in Cystic Fibrosis

Adherence to prescribed therapies is a central determinant of clinical outcomes in patients with CF [29]. However, sustaining consistent engagement with complex daily regimens remains challenging, particularly during childhood and adolescence. Increasing attention has been directed toward psychological factors that may influence adherence, with depression emerging as one of the most consistently associated correlates [29,30,31].

Evidence from multicentre prospective studies supports this association. In a longitudinal study conducted across 14 CF referral centres in Brazil, depressive symptoms were identified in approximately 15–19% of adolescents with CF, while nearly two-thirds reported low treatment adherence [32]. Both depression and anxiety demonstrated significant inverse correlations with adherence at two assessment points, indicating that higher symptom burden was associated with poorer adherence over time.

Findings from paediatric cohorts using validated adherence measures further reinforce these observations. In a study of children and adolescents aged 7–17 years, 29% exhibited elevated depressive symptoms, which were independently associated with lower adherence to airway clearance therapy after adjustment for demographic factors (r = −0.34, *p* = 0.02) [29]. Depressive symptoms were also linked to poorer parent–child relational security, and weaker relational security was itself associated with reduced adherence, highlighting the potential influence of family context on adherence behaviours in younger patients [29].

Additional insight is provided by retrospective clinical data comparing youth with CF diagnosed with a depressive disorder to matched non-depressed controls [33]. Although adherence was not directly measured, depressed patients experienced substantially higher hospitalisation rates and markedly greater healthcare costs despite comparable baseline lung function [33]. Together, these findings suggest that depressive symptoms are associated with clinically meaningful adherence-related consequences in CF. Most available evidence is observational and relies on symptom screening rather than diagnostic assessment. Nonetheless, the consistency of findings across prospective, cross-sectional, and retrospective designs supports depression as a clinically relevant factor associated with nonadherence in CF [29,32,33].

Following the introduction of ETI, the relationship between mental health and treatment adherence may be evolving. Although modulator therapy has reduced symptom burden and simplified some aspects of disease management, emerging evidence suggests that adherence patterns may change as patients adjust to improved health status. Some individuals report modifying or discontinuing inhaled therapies after ETI initiation, sometimes without medical supervision [28,34]. Whether depression continues to independently predict non-adherence in the modulator era, as consistently demonstrated in pre-modulator cohorts, requires further prospective investigation.

### 3.4. Depression and Survival in Cystic Fibrosis

The potential impact of depression on survival in CF has received less attention than its effects on clinical outcomes and quality of life. However, evidence from large observational cohorts suggests that depression may have prognostic relevance beyond established markers of disease severity [35].

In a cohort of over 1000 untransplanted adolescents and adults with CF followed for five years, depressive symptoms—present in approximately one quarter of participants—were associated with nearly a twofold increase in mortality risk in unadjusted analyses [35]. Although the strength of this association was attenuated after adjustment for clinical factors, depression remained independently associated with mortality among adults. The highest risk was observed in individuals with severe depressive symptoms, indicating a severity-dependent relationship. In contrast, anxiety was not associated with mortality despite its higher prevalence [35].

Causality cannot be established from observational data. Nevertheless, these findings suggest that depression may function as a clinically meaningful prognostic factor rather than solely as a consequence of advanced disease [35]. As survival in CF continues to improve, recognising and addressing depression may have important implications for long-term outcomes and risk stratification [19].

With ETI substantially improving survival trajectories for many individuals with CF, the prognostic relevance of depression in the current era remains uncertain. Registry-based post-authorisation safety data indicate that population-level depression prevalence has not increased following ETI initiation [19]. However, no longitudinal study has yet examined whether the association between depression and mortality previously reported in CF populations persists or is modified in ETI-treated cohorts. Clarifying this relationship will be an important priority for future longitudinal research as modulator-treated populations are followed over longer time horizons.

## 4. Screening for Depression and Anxiety in Cystic Fibrosis

The systematic identification of depression and anxiety has become a central component of comprehensive CF care, reflecting a broader shift toward integrating mental health within chronic disease management. Accumulating evidence has demonstrated that psychological distress is common in individuals with CF and their caregivers and is associated with clinically meaningful outcomes [7,12] including poorer adherence [29,30,31], increased healthcare utilisation [33], and adverse long-term trajectories [35]. Reliance on clinical impression alone has proven insufficient for detecting depression and anxiety in this population, prompting the development of standardised screening approaches designed for routine clinical use [36,37]. However, these screening recommendations were largely developed and validated in pre-modulator populations, and their performance and clinical thresholds in individuals receiving highly effective CFTR modulator therapy remain less clearly defined.

In recognition of these needs, the International Committee on Mental Health in Cystic Fibrosis (ICMH) published consensus recommendations for screening in 2016 [37]. These recommendations were jointly led by the Cystic Fibrosis Foundation (CFF) and the European Cystic Fibrosis Society (ECFS). These guidelines emphasise that screening should not be implemented in isolation but embedded within defined care pathways capable of providing timely assessment, referral, and appropriate treatment.

Before initiating routine screening, CF centres are advised to ensure that trained personnel with expertise in mental health assessment and management are available, such as licensed social workers, psychologists, psychiatrists, or other appropriately trained clinicians. Centres should also develop educational materials explaining the purpose and implications of screening and establish referral pathways within the hospital and the surrounding community. In addition, explicit protocols for responding to suicidal ideation must be in place prior to implementation to ensure patient safety [37].

Screening recommendations are stratified by developmental stage. For children with CF aged 7–11 years, the ICMH does not recommend routine questionnaire-based screening [37]. Instead, targeted clinical evaluation is advised when risk indicators for psychological distress are present including behavioural concerns, emotional difficulties, or elevated caregiver distress. This approach reflects developmental considerations and the limited validation of self-report screening tools in this age group.

For adolescents and adults with CF aged 12 years and older, the ICMH recommends annual screening for both depression and anxiety during periods of clinical stability [37]. Age-specific screening strategies, including target populations, screening frequency, and recommended instruments, are summarised in Table 2. The preferred instruments were selected based on their brevity, validity, and feasibility for use in routine clinical settings. Importantly, any positive screen should prompt further clinical assessment by an appropriately trained provider before treatment decisions are made.

Screening recommendations also extend to caregivers, acknowledging their central role in CF management and the interdependence between patient and caregiver mental health [37]. Identification of psychological distress in caregivers provides an opportunity for early support and may positively influence family functioning and patient outcomes [38,39].

Collectively, these recommendations represent an effort to standardise mental health screening in CF, addressing the previously wide variability in tools and practices across centres internationally [18]. Their successful implementation depends not only on the selection of validated instruments but also on the availability of trained personnel, clear referral pathways, and defined procedures for managing positive screens, including suicidal ideation. Ongoing evaluation of implementation processes and resource adequacy remains essential to ensure that screening leads to meaningful improvements in clinical care rather than functioning as an isolated or purely administrative exercise [40].

## 5. Treatment Approaches for Depression and Anxiety in Cystic Fibrosis

The management of depression and anxiety in CF requires a structured, evidence-based approach that accounts for the chronic, progressive nature of the disease, the substantial treatment burden, and the close interdependence between psychological well-being and clinical outcomes [41]. International guidelines emphasise that treatment should be guided by clinical assessment following systematic screening and delivered within multidisciplinary CF care models capable of providing longitudinal follow-up. A range of therapeutic strategies has therefore been proposed, spanning psychological interventions, pharmacological treatment, digital and telehealth-based approaches, and stepped-care frameworks designed to match treatment intensity to symptom severity and available resources.

### 5.1. Cognitive–Behavioural Therapy Interventions in Cystic Fibrosis

Cognitive–behavioural therapy (CBT) is the most extensively studied psychological intervention for depression and anxiety and is recommended as a core treatment modality for individuals with CF who exhibit clinically significant symptoms. Despite strong guideline endorsement, access to CBT tailored to the CF population remains limited [42]. Structural barriers including inadequate insurance coverage or financial support, limited availability of mental health professionals with CF-specific expertise, insufficient understanding of CF within community mental health services, and long waiting lists continue to hinder the implementation of evidence-based psychological care within routine CF practice [42].

These challenges are reflected in both caregiver and healthcare professional perspectives. In a large survey of 1454 caregivers, substantial gaps were reported in the training and resources needed to deliver guideline-concordant mental healthcare [18]. Similarly, surveys of CF healthcare professionals indicate that nearly half would welcome formal CBT training, highlighting workforce limitations as a major modifiable barrier [18].

Within a stepped-care framework, international guidelines recommend monitoring, psychoeducation, and supportive interventions for individuals with mild symptoms, with structured evidence-based therapies reserved for moderate to severe depression or anxiety [43]. CBT targets maladaptive cognitive and behavioural patterns, enhances coping skills, and supports emotional regulation, making it well-suited to the chronic and treatment-intensive context of CF. Evidence from chronic disease populations consistently demonstrates its benefit for emotional well-being, health-related quality of life, and symptom reduction [44,45].

Although CF-specific randomised trials remain limited, adaptations of CBT for CF have shown promise. An eight-session CF-specific CBT intervention designed for delivery within multidisciplinary CF teams demonstrated good feasibility and acceptability [46]. CBT-informed behavioural interventions targeting specific domains, such as sleep disturbance, have also shown feasibility in children and adolescents with CF, suggesting potential roles in both prevention and treatment.

CBT-based interventions have also been extended to caregivers, reflecting growing recognition of the interdependence between patient and caregiver mental health [47]. Web-based CBT-informed programmes for parents of children with CF have demonstrated clinically meaningful reductions in anxiety and depressive symptoms, with sustained improvements in quality of life at follow-up [47]. Collectively, available evidence supports CBT as a cornerstone psychological intervention in CF while highlighting ongoing needs related to workforce training and implementation. In the modulator era, improvements in physical health may be accompanied by new psychosocial challenges. These may include identity adjustment, changes in illness-related roles, and the need to recalibrate life expectations. As a result, the targets and content of CBT interventions may require adaptation to address emerging psychological needs in individuals receiving highly effective CFTR modulator therapy [15].

### 5.2. Telemedicine and Digital Interventions in Cystic Fibrosis

Telemedicine—also referred to as telehealth or electronic health (e-health)—has emerged as a critical modality for delivering psychological care in CF, particularly in the context of infection-control requirements, geographic barriers, and treatment burden [48]. In CF, telemedicine has been increasingly adopted to enhance access to care, facilitate monitoring of respiratory and biometric parameters, and maintain continuity of multidisciplinary support.

Evidence indicates that telemedicine enables home monitoring of clinical parameters while also facilitating access to psychological support [49]. The coronavirus disease 2019 (COVID-19) pandemic accelerated the adoption of telehealth globally and highlighted its value in maintaining psychological support for individuals with CF during periods of restricted access to in-person care [50]. Significantly, the relevance of telemedicine for mental healthcare in CF extends beyond the pandemic context and should be considered a core component of future care models.

Several telehealth psychological interventions have demonstrated feasibility and benefit. During the COVID-19 pandemic, a CF centre in Rome implemented a telehealth psychological support program for patients and caregivers that included four CBT-informed sessions [51]. Baseline screening identified high levels of depression and anxiety (71%). Following the intervention, depressive symptoms improved significantly in both patients and caregivers, although anxiety symptoms showed less change.

Remote delivery of structured psychological therapies has also shown promise. Acceptance and Commitment Therapy (ACT) [52], which has been adapted for telehealth, was evaluated in adults with CF, demonstrating high acceptability, strong completion rates, and clinically meaningful reductions in psychological distress, with outcomes comparable to in-person delivery [53]. Similarly, telehealth-based CBT interventions, including the Coping and Learning to Manage Stress with CF (CALM) program, have demonstrated improvements in depression, anxiety, coping, and health-related quality of life in pilot trials [54,55,56,57]. These programmes explicitly address CF-specific stressors such as medical procedures, hospitalisations, uncertainty about illness trajectory, and survivor’s guilt.

Beyond structured psychotherapy, mobile health (mHealth) interventions represent an emerging digital strategy for supporting psychological well-being in CF, particularly among adolescents [58]. Evidence suggests that mHealth tools may enhance enjoyment, social engagement, and emotional expression while reducing pain, anxiety, distress, and stress in paediatric CF populations. Collectively, telehealth and mHealth interventions offer a valuable opportunity to overcome barriers to mental healthcare access, reduce healthcare burden, and integrate psychological support more seamlessly into CF care pathways. These promising early results lay the groundwork for broader integration of digital mental health tools in standard CF care.

### 5.3. Narrative Medicine in Cystic Fibrosis

Narrative medicine has emerged as a complementary therapeutic and educational approach in the management of chronic illness, with growing relevance to CF [41]. This approach emphasises structured listening and an exploration of patients’ illness narratives to enhance understanding of lived experience, promote engagement, and support psychosocial adjustment [59].

Illness narratives enable individuals to organise experiences, construct meaning, and process emotional challenges associated with chronic disease [60]. In other chronic conditions, including inflammatory bowel disease, diabetes, and heart disease, narrative-based interventions have been associated with improved disease understanding, emotional adaptation, and self-management behaviours. Exposure to positive illness narratives has also been linked to increased hope and emotional well-being [60,61].

In paediatric and family-centred care, narrative-based methods have demonstrated particular value. Group narrative interventions with parents of children with chronic illness have facilitated emotional expression and strengthened coping resources. Narrative and play-based approaches have also been used to explore the experiences of young children with CF, providing insights not readily captured through conventional assessments [62]. These approaches offer insights into children’s internal experiences that may not be accessible through conventional assessment methods and may support more developmentally sensitive care.

Within CF-specific settings, narrative medicine remains underexplored but shows emerging promise. Preliminary work using narrative-based and metacognitive approaches has reported improvements in anxiety and emotional functioning, with high acceptability among patients and caregivers [63]. While current evidence is limited, these approaches may offer meaningful adjunctive benefits when integrated alongside established psychological treatments.

### 5.4. Counselling in Cystic Fibrosis

Counselling individuals with CF presents distinctive clinical and developmental challenges that require specialised expertise beyond general mental health practice [37,42]. The chronic, progressive nature of CF, combined with intensive treatment demands and recurrent medical stressors, places patients, particularly children and adolescents, at increased risk of psychological distress. Effective counselling therefore depends on the clinicians’ ability to integrate strong communication skills, active listening, and disease-specific knowledge to engage patients meaningfully across developmental stages. Mental health professionals working in CF care must be sensitive to the interplay between physical illness, emotional well-being, and psychosocial development [41].

Adolescence brings unique psychological challenges in CF, with notable gender-specific differences. Research exploring the transition from adolescence to adulthood in CF has highlighted important gender-specific differences in psychological experience [64]. Female adolescents report higher levels of depressive symptoms, negative body image, and fears related to CF-associated complications such as diabetes. In contrast, male adolescents tend to demonstrate greater behavioural independence and disclose emotional concerns less readily, often only as disease burden increases [64]. These findings underscore the need for counsellors to attend closely to developmental timing, gendered illness experiences, and evolving illness acceptance when working with young people with CF.

Counselling for children and adolescents with CF must be tailored to developmental stage and illness context. Young patients often face challenges related to emotional regulation, identity development, and social functioning alongside disease progression [65]. Counsellors should remain attentive to illness acceptance, treatment-related distress, and emerging mental health concerns over time.

Family involvement is a central component of counselling in CF and should be integrated from the point of diagnosis onward. Chronic illness exerts a profound impact on family systems, with parents often assuming primary responsibility for treatment management while coping with their own psychological distress [66]. Counselling interventions should therefore extend beyond the individual patient to address family functioning, parental coping, and the balance between fostering resilience and supporting autonomy. In some cases, parents may benefit from individual counselling to strengthen their capacity to support their child effectively. Additionally, counsellors may play an important role in supporting patients and families during key clinical transitions, including the initiation of CFTR modulator therapies, where education, expectation management, and emotional processing are critical [8].

### 5.5. Pharmacological Interventions for Depression and Anxiety in Cystic Fibrosis

Pharmacological treatment plays a targeted and complementary role in the management of depression and anxiety in CF, particularly when symptoms are moderate to severe or when psychological interventions are unavailable, declined, or insufficiently effective [67]. The ICMH consensus emphasises that antidepressant therapy should generally be delivered as part of a comprehensive treatment plan that includes psychological interventions, with combined approaches preferred in cases of severe depression. In contrast, pharmacotherapy may be considered earlier in the treatment course for moderate depression or moderate-to-severe anxiety if access to psychotherapy is limited [37,67].

A stepped-care pharmacological approach aligned with symptom severity and adapted from the International Committee on Mental Health in Cystic Fibrosis (ICMH) recommendations is summarised in Table 3. This framework guides when pharmacological treatment is not indicated, when it may be considered, and when initiation is recommended, while emphasising monitoring and safety across all severity levels.

Selective serotonin reuptake inhibitors (SSRIs) are recommended as first-line pharmacological agents for adolescents and adults with CF requiring medication treatment for depression and/or anxiety [37]. Among the available agents, citalopram, escitalopram, sertraline, and fluoxetine are preferred due to established efficacy, favourable tolerability, and a lower risk of clinically significant drug–drug interactions. Their effectiveness across both depressive and anxiety disorders is particularly relevant given the high degree of symptom comorbidity in CF.

Prescribing and dose titration require careful individualisation. CF-related alterations in gastrointestinal absorption, hepatic metabolism, nutritional status, and organ function may influence pharmacokinetics and contribute to substantial inter-individual variability [68]. Clinicians should monitor treatment response and adverse effects closely, with dose adjustment as required. When available, therapeutic drug monitoring may support clinical decision-making, although routine use is not universally necessary [68].

Vigilance for drug–drug interactions is essential, and maintaining an up-to-date medication history is crucial. Enzyme-inducing CFTR modulators such as lumacaftor may reduce SSRI exposure and necessitate higher doses to achieve effect [69]. Conversely, co-administration of linezolid with serotonergic antidepressants should generally be avoided unless no alternatives exist due to the risk of serotonin syndrome [70]. SSRIs can also modestly prolong the QT interval, especially citalopram; combining them with other QT-prolonging medications warrants careful monitoring such as periodic ECG and electrolyte checks. For acute, procedure-related anxiety that is unresponsive to behavioural strategies, a short-term course of a benzodiazepine such as lorazepam may be considered with strict attention to respiratory status, sedation risk, and duration of use [37].

Despite the availability of evidence-based psychological and pharmacological interventions, translating these approaches into routine CF care remains challenging [18,40]. Many CF centres lack embedded mental health professionals, and access to trained providers with CF-specific expertise is often limited. Workforce shortages, competing clinical priorities, and variability in healthcare resources across countries can further constrain implementation [40]. Practical strategies to address these gaps may include integrating mental health professionals within multidisciplinary CF teams, expanding training for existing team members in basic psychological support, and utilising telehealth platforms to extend access to evidence-based interventions such as CBT [50,53]. These approaches may be particularly relevant in healthcare settings with limited specialist resources.

## 6. Special Populations and Contexts

Research has clarified how common depression and anxiety are in CF and how they affect health and care. However, mental health risk does not affect all individuals or settings in the same way. Certain life stages and clinical contexts place people with CF and their families under added emotional strain. Adolescence and the move to adult care, caregiving roles, and pre- and post-transplant periods are times when usual screening and treatment approaches may need adjustment. Examining these contexts more closely helps explain why some patients and families remain vulnerable despite medical progress and highlights the need for tailored psychosocial care.

### 6.1. Adolescents and Transition to Adult Care

Adolescence represents a particularly vulnerable period for individuals with CF, as major developmental, social, and health-related transitions occur simultaneously [16]. During this stage, young people increasingly assume responsibility for daily treatment regimens while navigating education, employment planning, peer relationships, and the transition from paediatric to adult CF care [37]. These overlapping demands can place substantial strain on psychological well-being, particularly for individuals who struggle with treatment self-management or who fear disease progression [16].

Prior to the introduction of highly effective modulator therapy (HEMT), adolescents and young adults with CF consistently demonstrated elevated rates of anxiety and depression. A meta-analysis reported that approximately 18.7% met the criteria for depressive or anxiety disorders, a prevalence at least twice that observed in the general population [14]. These findings established adolescence as a high-risk period for mental health difficulties in CF.

The advent of elexacaftor–tezacaftor–ivacaftor (ETI) has fundamentally altered disease trajectories for many adolescents and young adults. While the physical benefits of ETI are well-documented, its psychosocial impact during this developmental stage is more complex. Adolescents now anticipate longer and healthier lives, reshaping expectations around independence, education, career planning, relationships, and family life. For many, this shift is experienced as profoundly hopeful. However, it also challenges long-standing perceptions of CF identity and disrupts established coping narratives [8,71,72].

Qualitative studies describe ETI as “life-changing” or “liberating”, with adolescents reporting improvements in energy, daily functioning, and optimism. Many express feeling “normal again” and describe new aspirations that were previously viewed as unattainable. At the same time, this rapid change can provoke emotional adjustment difficulties. Some adolescents report identity disruption as they attempt to redefine themselves in the absence of long-standing illness-related limitations. Persistent grief and anxiety also remain common, particularly in relation to the loss of peers, uncertainty about long-term treatment durability, and fears of treatment failure or discontinuation [71,72].

Quantitative data reflect this heterogeneity. Longitudinal cohort studies show that many adolescents with elevated anxiety or depressive symptoms prior to ETI initiation improve to normative levels within 12–18 months [71]. In contrast, other studies reported no significant change in average mental health scores following treatment initiation and identified a smaller subgroup who develop new-onset anxiety or depressive symptoms post-ETI [20]. These findings highlight that improved physical health does not uniformly translate into psychological resilience during a developmental stage already marked by identity formation and social transition.

Clinically, these data underscore the need for proactive, developmentally appropriate psychosocial care. Mental health screening and counselling for adolescents with CF should explicitly address peer loss, identity change, emerging independence, and future uncertainty, rather than assuming that physical improvement alone mitigates emotional vulnerability [8].

### 6.2. Caregivers’ Mental Health

Caregivers of individuals with CF experience substantial and sustained psychological burden. The responsibilities of daily treatment management, ongoing vigilance for clinical deterioration, and uncertainty regarding long-term outcomes contribute to chronic emotional strain [39]. Both quantitative and qualitative studies consistently demonstrate high rates of anxiety and depression among caregivers [16].

Meta-analytic evidence indicates that approximately one-third of caregivers screen positive for depressive symptoms and over one-third for anxiety. Greater disease severity is associated with higher caregiver burden and worse psychological outcomes. Commonly reported stressors include fear for the child’s health, guilt related to genetic inheritance, and exhaustion related to the cumulative demands of care [39]. Importantly, caregiver psychological distress has been associated with poorer treatment adherence and adverse patient outcomes, highlighting the interdependent nature of family well-being in CF [29,33].

Recognising this burden, international CF guidelines recommend routine mental health screening for at least one primary caregiver of children and adolescents with CF [37]. The introduction of HEMT has altered the caregiving experience for many families. Improvements in respiratory symptoms and reductions in treatment burden have been associated with reclaimed personal time, improved daily routines, and enhanced family quality of life [72]. Emerging evidence suggests that these clinical gains translate into measurable improvements in caregivers’ physical quality of life and perceived daily burden [72].

However, psychological outcomes are more variable. While some caregivers report reduced distress, anxiety and depressive symptoms often persist or evolve rather than fully resolve [39]. Emotional adjustment may involve ongoing uncertainty, hypervigilance, or difficulty relinquishing long-standing caregiving roles despite improved clinical stability [11].

A particularly vulnerable subgroup includes caregivers of individuals who remain ineligible for HEMT due to genotype, age, or intolerance. For these families, psychological burden may be intensified by feelings of inequity, disappointment, and grief related to missed therapeutic opportunities [11,72]. The ongoing intensity of care, combined with awareness of transformative treatments available to others, creates a distinct psychosocial context that requires tailored support and targeted interventions.

### 6.3. Pre- and Post-Transplant Contexts

For some individuals with CF, especially before the modulator era, disease progression led to end-stage organ failure requiring transplantation, most commonly a double lung transplant, and sometimes liver transplant. Historically, CF was the third most common indication for lung transplantation [73]. The period leading up to a transplant—when a patient is sick enough to be listed for an organ—represents a peak of psychological vulnerability for both patients and their families. This phase is fraught with chronic stress and uncertainty. Patients on the lung transplant waiting list commonly experience intense anxiety, depression, and existential distress. Studies from the pre-HEMT era reported that between 20% and 47% of CF patients evaluated for transplant showed clinically significant symptoms of anxiety or depression [74,75]. Key stressors include the constant tension of waiting for a suitable donor match and the fear that time will run out—that the transplant might come too late or not at all [76]. Every day on the waitlist carries uncertainty about survival, which can be emotionally exhausting. This psychological distress often also feeds into physical well-being; heightened anxiety and depression have been associated with an increased perception of physical symptoms or even actual health deterioration in patients awaiting transplant [74,75].

In light of these challenges, it is vital for CF and transplant teams to address mental health needs during this pre-transplant period. Unmanaged distress can negatively impact treatment adherence and may worsen outcomes. In fact, research has shown that anxiety and other psychiatric symptoms in transplant candidates are linked to poorer medication adherence and potentially lower post-transplant survival rates [76]. Early identification and treatment of psychological distress before transplant is therefore not just about improving mental health. In fact, it can be life-saving from a medical standpoint by reducing the risk of complications or graft failure. Professional guidelines underscore this point: organisations like the CFF recommend that transplant centres routinely assess and support patients’ mental health during the evaluation and waiting period [77]. Interventions such as counselling, peer support, or psychiatric care when needed can help patients and families cope with the wait and improve readiness for transplant [76].

The landscape of transplantation in CF has shifted dramatically in the era of highly effective modulators. With many patients experiencing significant lung function improvement on drugs like ETI, far fewer people with CF are progressing to the point of needing a lung transplant. For example, in the United States, the annual number of lung transplants performed in CF dropped substantially, falling from 243 to 56.7 post-ETI [78]. This is an astonishing change over just a few years. It means that the current population of CF patients who still require transplants is smaller and often comprised of those with special circumstances, primarily individuals who cannot take modulators due to non-eligible mutations or other issues, or those for whom the modulator therapy is not sufficiently effective [78]. This new reality carries its own psychosocial implications. Patients and families who once braced for transplant may find their trajectory altered by modulators, which is undoubtedly a relief but can also be disorienting. Being delisted from a transplant waiting list because of health improvement is a remarkable medical victory, yet some patients have described an unexpected psychological impact. Those who had mentally prepared themselves for end-stage disease and the possibility of transplantation must now adjust to a new future that is neither the grave scenario they anticipated nor a cure. They move from the immediacy of life-threatening illness to a state of improved but still chronic health, which can leave them feeling unmoored or uncertain about their identity and plans [8]. In other words, they need to redefine their sense of self and hopes for the future outside the shadow of an imminent transplant. On the other hand, patients who do still face transplant in the modulator era may carry an added sense of being “left behind” medically, which can heighten feelings of unfairness or despair [11]. Care teams should be mindful of these complex emotions and provide counselling or peer connections for both groups, those coming off the transplant list and those remaining on it [37].

For individuals who do undergo lung transplantation, the post-transplant period generally brings substantial improvements in physical health and overall quality of life. Multiple studies have found that on average, CF patients experience significant boosts in health-related quality of life after lung transplant compared to their pre-transplant status [79]. Anxiety and depression levels tend to drop following transplant, and many recipients maintain these mental health gains in the long-term. In fact, up to five years after surgery, many CF lung transplant recipients report quality-of-life scores approaching those of the general population [80]. Freed from the respiratory failure of end-stage CF, patients can often breathe easier, gain weight, and participate in activities that were impossible before, all of which contributes positively to mood and well-being.

However, despite these overall positive trends, persistent psychosocial challenges remain. A significant minority of post-transplant patients continue to experience mental health difficulties that require attention. Recent surveys indicate that roughly 20–30% of CF lung transplant recipients still exhibit elevated symptoms of depression or anxiety at any given time after the surgery [76]. Additionally, around 1 in 8 (approximately 12%) have reported clinical signs of post-traumatic stress disorder (PTSD) related to the trauma of undergoing transplantation and the medical ordeals surrounding it [76]. When compared to healthy individuals, CF patients post-transplant report lower satisfaction in certain quality-of-life domains, particularly in areas like career or employment concerns, worries about the future, and body image issues. For instance, coping with surgical scars or the long-term effects of medication, and interpersonal relationships [80]. These enduring issues highlight that transplant is not a psychological “cure-all”; even when lungs are replaced and breathing improves, the person must still contend with a new set of life adjustments and uncertainties.

One crucial concern is that unresolved mental health symptoms after transplant can have medical consequences. Anxiety or depression in a transplant recipient may undermine the rigorous adherence required for post-transplant care. For instance, patients with high anxiety might be less consistent in taking their immunosuppressive medications or in following complex medical instructions, which can increase the risk of rejection or other complications [76]. Therefore, ongoing mental health support is an integral component of post-transplant care. Standard CF care guidelines already call for annual depression and anxiety screening in all patients aged 12 and over and their caregivers, but in the transplant context, this monitoring should be even more frequent and finely tuned. Transplant teams, in collaboration with CF care providers, should implement more intensive psychological screening and interventions during the critical post-surgery period and beyond [77].

In summary, whether in the pre-transplant waiting phase or years into post-transplant recovery, attention to mental health is paramount. The modulator era has reduced the overall need for transplantation and changed patient expectations, but it has not eliminated the psychosocial complexities of these scenarios. Tailoring mental health services to meet the needs of patients in high-stress transitions such as adolescence, major treatment advances, or transplantation, is essential to ensure that improved physical health is accompanied by optimal emotional well-being [8].

## 7. Implementation Challenges

Despite broad consensus that mental health should be fully integrated into the CF multidisciplinary care model, implementation remains inconsistent [18,37]. Although international CF consensus guidelines call for routine annual depression and anxiety screening, translating these recommendations into consistent high-quality practice has proven difficult.

### 7.1. Barriers to Mental Health Integration in CF Centres

A major implementation challenge identified in evaluation studies is the gap between symptom identification and access to timely psychological intervention [18,37]. While standardised mental-health screening has been adopted by many CF centres following the publication of international guidelines, positive screening results do not consistently lead to timely psychological assessment or treatment. In some programmes, screening initiatives have outpaced the development of referral pathways and follow-up services, leaving identified psychosocial needs unmet. Care delivery may also remain fragmented in some health systems, with mental-health services provided outside the CF clinic and reliant on external referrals. Such fragmentation can limit continuity of care and contributes to delays in intervention, particularly during periods of heightened psychological vulnerability, including transition from paediatric to adult care and pre-transplant evaluation [8,37]. For individuals already managing substantial treatment burden, additional appointments in separate services may further hinder engagement and follow-through.

Importantly, the psychosocial landscape has evolved in the modulator era. As discussed earlier, some patients experience new emotional challenges and identity adjustments when their disease trajectory rapidly improves such as feeling unmoored after coming off the transplant list or anxiety over long-term treatment durability. Such findings underscore the need for flexible, CF-specific mental health support rather than generic referral models [8].

Addressing these barriers requires organisational strategies that embed mental healthcare within routine CF services rather than relying solely on external referral pathways. Integrating mental health professionals into multidisciplinary CF teams, where feasible, can facilitate timely assessment and intervention [40]. In centres where dedicated specialists are unavailable, structured referral protocols and collaboration with community mental health providers may help reduce delays in care and improve the continuity of psychological support [18].

### 7.2. Workforce and Resource Limitations

Limited availability of trained mental-health professionals embedded within CF multidisciplinary teams has been identified as a key constraint in implementation evaluations [18]. As noted earlier, a major barrier to providing psychosocial care is the shortage of dedicated CF mental health specialists. Many centres lack embedded psychologists/psychiatrists, overburdening the existing team and limiting the delivery of timely therapy.

Resource limitations are particularly evident in transplant-related care, where psychological distress has been consistently associated with adherence challenges and clinical outcomes [76]. Despite consensus recommendations emphasising the importance of mental-health assessment and support during transplant referral and evaluation, access to intensive psychosocial care remains inconsistent, potentially contributing to difficulties in adherence and post-transplant management [76].

Another implementation gap is in caregiver support. Although guidelines call for screening at least one primary caregiver, many centres lack the infrastructure for providing interventions when a caregiver screens positive. This is especially concerning for caregivers of patients not eligible for modulators—a group identified previously as carrying persistent high burden despite recent treatment advances. Without dedicated follow-up resources, these caregivers’ needs often go unmet [14,81].

Expanding workforce capacity may also require innovative approaches to service delivery. Training existing CF team members such as nurses, social workers, or allied health professionals in basic psychosocial support and screening follow-up may help bridge gaps in care [40]. In addition, telehealth-based psychological services may offer a feasible strategy to extend access to evidence-based interventions, particularly in regions where specialised CF mental health expertise is limited [50,53].

### 7.3. Need for Standardised Care Pathways

The observed variability in mental-health implementation across CF centres highlights the absence of standardised, actionable care pathways that consistently link screening outcomes to intervention and follow-up. Evaluations of guideline implementation describe substantial heterogeneity in referral thresholds, response timelines, and access to CF-informed psychological services, contributing to inequities in care delivery [37].

As CF care continues to evolve in the post-modulator era, mental-health pathways must also adapt. Emerging evidence suggests that annual screening alone may be insufficient for individuals navigating high-risk clinical contexts, such as adolescence, transplant listing, delisting following clinical improvement, or periods of significant treatment transition [8,40]. Screening strategies therefore require contextualisation and intensification according to clinical risk.

Importantly, standardised pathways should explicitly link psychological assessment to medical outcomes, particularly treatment adherence. Anxiety and depressive symptoms have been consistently associated with compromised adherence to complex CF and post-transplant treatment regimens [29,76]. Without integrated care pathways that translate screening results into coordinated psychological intervention, the potential clinical benefits of routine mental-health screening may not be fully realised [76].

Establishing standardised care pathways that clearly link screening results to defined clinical actions may help translate guideline recommendations into routine practice [18,40]. Such pathways could include predefined referral thresholds, structured follow-up timelines, and the integration of mental health monitoring within existing CF registry systems to support quality improvement efforts [18].

## 8. Implications for Research and Care

Addressing the challenges above will require both new research and changes in care delivery. Key priorities include conducting longitudinal studies, tailoring approaches to different cultural contexts, leveraging digital health tools, and integrating mental health data into CF registries [15].

### 8.1. Longitudinal Studies on Mental Health Outcomes and Survival

Given the predominance of cross-sectional studies in this field and the limited data on true incidence noted earlier, there is a clear need for longitudinal research to track how anxiety and depression evolve over the CF lifespan. Future studies should follow individuals across key stages such as adolescence, adulthood, post-CFTR modulator initiation, and transplantation to determine if and how psychosocial trajectories correlate with clinical outcomes and survival.

Future research should also examine whether persistent or recurrent symptoms of anxiety and depression are associated with long-term clinical outcomes such as pulmonary decline, hospitalisation, transplant outcomes, and survival. Extended follow-up is particularly important in the modulator era, where improvements in physical health may alter psychological trajectories and traditional predictors of prognosis [6,8]. Such data would help clarify whether mental health outcomes function primarily as markers of disease burden or as modifiable contributors to long-term risk.

### 8.2. Culturally Adapted Screening and Interventions

Commonly used screening tools like the PHQ-9 and GAD-7 were originally developed in Western populations. Their accuracy and relevance in other cultural and linguistic settings are still uncertain. Cross-national studies, including large CF cohorts, have shown substantial variation in reported depression and anxiety rates across countries despite using identical instruments [6]. Other validation studies have found that these tools may function differently across languages or cultural groups. For example, some populations are more likely to express emotional distress through somatic symptoms, which may not be captured by standard screening items [82]. This mismatch can result in under recognition and delayed psychological support. Adaptation and revalidation are necessary to ensure that screening tools truly reflect the needs of diverse CF populations.

The same principle applies to treatment. Psychosocial interventions should reflect the daily realities of patients, how families function, what people believe about illness, and what kind of care is truly available. In places where mental health carries stigma, or where therapy resources are limited, interventions must be both sensitive and practical. When care models are built around what matters to patients and their families, engagement tends to improve as well as the outcomes [40].

### 8.3. Expanding Digital Mental-Health Solutions

Building on early telehealth successes in CF care, future efforts should rigorously evaluate digital mental-health solutions. Remote screening, teletherapy, and mobile interventions have shown promise in other chronic diseases and in pilot CF studies, but their long-term impact in CF remains under-explored [58]. Moving forward, studies should assess how digital mental health tools perform when integrated into routine CF care. Rather than evaluating an app or telehealth program in isolation, research should determine whether incorporating these tools into standard practice improves outcomes such as symptom severity, adherence, or patient engagement, and how well they fit into existing clinic workflows [18]. It will also be important to establish guidelines on which patients can be managed adequately through telehealth and which require in-person support, ensuring that technology complements but does not replace face-to-face mental health services for individuals with more severe or complex needs [51].

### 8.4. Integrating Mental Health into CF Registries

National and international CF registries have become invaluable for understanding long-term disease outcomes and the impact of new therapies. However, most registries currently do not collect data on mental health [18,40]. Incorporating standardised mental health measures—for example, annual screening scores or documented diagnoses of depression/anxiety—into these registries represents an important future direction for both research and quality improvement.

Including mental health variables in CF registries would enable large-scale analyses of psychological symptom trends and their associations with clinical outcomes. For instance, researchers could examine how depression and anxiety levels change over time in the CF population and whether they predict outcomes like pulmonary exacerbations or treatment adherence. Registry data could also be used to evaluate the real-world implementation of mental health guidelines such as what proportion of patients who screen positive receive interventions and to identify subgroups of patients or caregivers at elevated risk of psychosocial distress. Achieving this integration will require consensus on appropriate measures, attention to patient privacy and data governance, and workflows that embed mental health data collection into routine care without placing undue burden on clinic teams [18,37].

By continuing to address these research gaps and implementation challenges, the CF community can work toward fully integrating mental health into standard CF care—ensuring that as medical treatments advance and extend lives, patients’ and families’ emotional well-being is also prioritised and supported.

## 9. Conclusions

Depression and anxiety are prevalent, clinically meaningful comorbidities in CF. It extends beyond psychological symptoms to influence treatment adherence, health-related quality of life, healthcare utilisation, and potentially survival. Available evidence suggests that these associations are not merely attributed to disease severity, supporting a bidirectional relationship between mental health and clinical outcomes. Despite substantial advances in CF care, including the introduction of highly effective CFTR modulator therapies, psychological vulnerability persists for many individuals and caregivers, particularly during key transitional and high-stress periods such as adolescence, transplant pathways, and caregiving roles.

Although international guidelines provide clear recommendations for mental health screening and stepped-care treatment in CF, implementation remains inconsistent. Persistent gaps exist between the identification of psychological distress and access to timely, CF-informed interventions. Future research should prioritise longitudinal studies, the development of context-sensitive care pathways, and the integration of mental health services into routine CF care to ensure that gains in physical health are accompanied by sustained psychological well-being.

## Figures and Tables

**Table 1:** Reported prevalence range of depressive and anxiety symptoms in people with cystic fibrosis and caregivers.

Population	Depression (%)	Anxiety (%)
Children and adolescents with CF	8–29	20–30
Adults with CF	13–33	28–33
Caregivers (primarily parents)	20–35	35–38

Data summarised from [6,7,14].

**Table 2:** Recommended screening approaches for depression and anxiety in cystic fibrosis.

Population	Age Group	Screening Strategy	Recommended Instruments
Children with CF	7–11 years	Targeted clinical evaluation when risk indicators are present	Clinical assessment by a trained mental health professional
Adolescents with CF	≥12 years	Annual screening during clinical stability	Patient Health Questionnaire-9 (PHQ-9) + Generalised Anxiety Disorder-7 (GAD-7)
Adults with CF	≥18 years	Annual screening during clinical stability	Patient Health Questionnaire-9 (PHQ-9) + Generalised Anxiety Disorder-7 (GAD-7)
Caregivers of children/adolescents with CF	Child age 0–17 years	Annual screening of at least one primary caregiver	PHQ-9 + GAD-7 or PHQ-8 + GAD-7 or PHQ-2 + GAD-2
Any positive screen	—	Requires further clinical assessment before treatment	Assessment by an appropriately trained provider
Children with CF	7–11 years	Targeted clinical evaluation when risk indicators are present	Clinical assessment by a trained mental health professional

Abbreviations: CF, cystic fibrosis; PHQ-9, Patient Health Questionnaire-9; PHQ-8, Patient Health Questionnaire-8; PHQ-2, Patient Health Questionnaire-2; GAD-7, Generalised Anxiety Disorder-7 scale; GAD-2, Generalised Anxiety Disorder-2 scale. Adapted from the International Committee on Mental Health in Cystic Fibrosis recommendations [37].

**Table 3:** Stepped-care pharmacological approach to depression and anxiety in cystic fibrosis (adapted from the ICMH Recommendations).

Symptom Severity (PHQ-9/GAD-7)	Primary Approach	Pharmacological Role	Key Considerations
No symptoms/Minimal (PHQ-9 or GAD-7: 0–4)	Routine screening, preventive support	Not indicated	Annual rescreening
Mild (PHQ-9 or GAD-7: 5–9)	Psychoeducation, supportive care, rescreen	Not routinely indicated	Monitor symptoms
Moderate (PHQ-9 or GAD-7: 10–14)	Evidence-based psychological intervention (CBT or IPT)	Consider an SSRI if psychotherapy is unavailable or ineffective	Monitor response and interactions
Severe depression (PHQ-9 ≥ 15)	Combined psychological therapy + SSRI	Initiate SSRI (citalopram, escitalopram, sertraline, fluoxetine)	Close monitoring, safety assessment
Severe anxiety (GAD-7 ≥ 15)	Exposure-based CBT	SSRI if CBT unavailable or ineffective	Avoid benzodiazepines long-term
Procedure-related anxiety	Behavioural strategies	Short-term lorazepam if needed	Respiratory risk, short duration

Abbreviations: CBT, cognitive behavioural therapy; CF, cystic fibrosis; GAD-7, Generalised Anxiety Disorder 7-item scale; ICMH, International Committee on Mental Health in Cystic Fibrosis; IPT, interpersonal therapy; PHQ-9, Patient Health Questionnaire-9; SSRI, selective serotonin reuptake inhibitor. Adapted from the International Committee on Mental Health in Cystic Fibrosis recommendations [37].

## Data Availability

No new data were created or analyzed in this study.

## Abbreviations

The following abbreviations are used in this manuscript:

CF	Cystic Fibrosis
ACT	Acceptance and Commitment Therapy
CBT	Cognitive Behavioural Therapy
CFF	Cystic Fibrosis Foundation
CFTR	Cystic Fibrosis Transmembrane Conductance Regulator
DSM-5	Diagnostic and Statistical Manual of Mental Disorders, Fifth Edition
HRQoL	Health-Related Quality of Life
PHQ-9	Patient Health Questionnaire-9
GAD-7	Generalised Anxiety Disorder-7
TIDES	International Depression and Anxiety Epidemiological Study
ICMH	International Committee on Mental Health
ETI	Elexacaftor/Tezacaftor/Ivacaftor
HEMT	Highly Effective Modulator Therapy
SSRI	Selective Serotonin Reuptake Inhibitor
